# Gel-Based 3D Food Printing for Dysphagia Management: Advances in Personalized Nutrition, Texture Control, and Clinical Translation

**DOI:** 10.3390/gels12040289

**Published:** 2026-03-29

**Authors:** Ming Yang, Keping Chen, Zhou Qin, Xujing Zhu, Yuqing Zhang, Zhikun Yang

**Affiliations:** 1 Agricultural Product Processing and Storage Lab, School of Food and Biological Engineering, Jiangsu University, Zhenjiang 212013, China; 2 Key Laboratory of Chinese Cuisine Intangible Cultural Heritage Technology Inheritance, Ministry of Culture and Tourism, College of Tourism and Culinary Science, Yangzhou University, Yangzhou 225127, China

**Keywords:** 3D food printing, gel-based inks, dysphagia, emulsion gels, personalized nutrition

## Abstract

Dysphagia and age-related oral processing limitations are rising with population aging and the growing burden of neurological diseases. Texture-modified diets remain the most common non-pharmacological intervention, yet conventional pureeing and thickening often yield meals with low visual appeal, variable textures, and diluted nutrient density, which contribute to reduced intake and malnutrition risk. Extrusion-based three-dimensional food printing, especially when combined with gel-derived edible inks, offers a digital route to standardize geometry, portioning, and texture while enabling individualized nutrition and sensory design. In the past three years, the field has progressed from simple single-ingredient pastes to engineered soft-matter systems including emulsion gels, high-internal-phase emulsion gels, Pickering-stabilized gels, bigels, and multi-material constructs enabled by dual and coaxial printing. These advances are underpinned by improved rheological windowing, microstructure engineering, and post-print gelation strategies such as ionic crosslinking, thermal setting, enzymatic bridging, and pH-triggered network formation. Meanwhile, dysphagia-oriented product development has matured from “shape recovery” demonstrations toward clinically relevant texture targets, leveraging the IDDSI tests to anchor swallowability. This review synthesizes the recent literature across materials science, food engineering, and clinical nutrition to connect gel microstructure to extrusion performance, post-processing stability, and oral processing outcomes that are relevant to older adults and dysphagia patients. We propose design principles for gel network selection, phase structuring, and process control that simultaneously satisfy print fidelity and swallowing safety targets.

## 1. Introduction

Population aging has shifted the central nutrition challenge from alleviating scarcity to delivering precision nutrition tailored to functional decline and clinical risk [1,2]. Many older adults experience a convergent burden of anorexia of aging, chronic low-grade inflammation, polypharmacy, compromised dentition, and neuromuscular deterioration, all of which impair oral processing. These changes increase vulnerability to malnutrition and sarcopenia and diminish physiological resilience during acute illness and rehabilitation [3,4]. Oropharyngeal dysphagia is a particularly high-impact contributor because it directly compromises swallowing safety and elevates the risk of aspiration, pneumonia, dehydration, and preventable hospitalizations. In clinical practice, texture modification remains the most common compensatory strategy because it is readily implemented and requires minimal specialized equipment [5]. However, this approach can be counterproductive when it reduces sensory enjoyment, suppresses appetite, or yields textures that vary unpredictably across caregivers and preparation settings.

**Figure 1:**
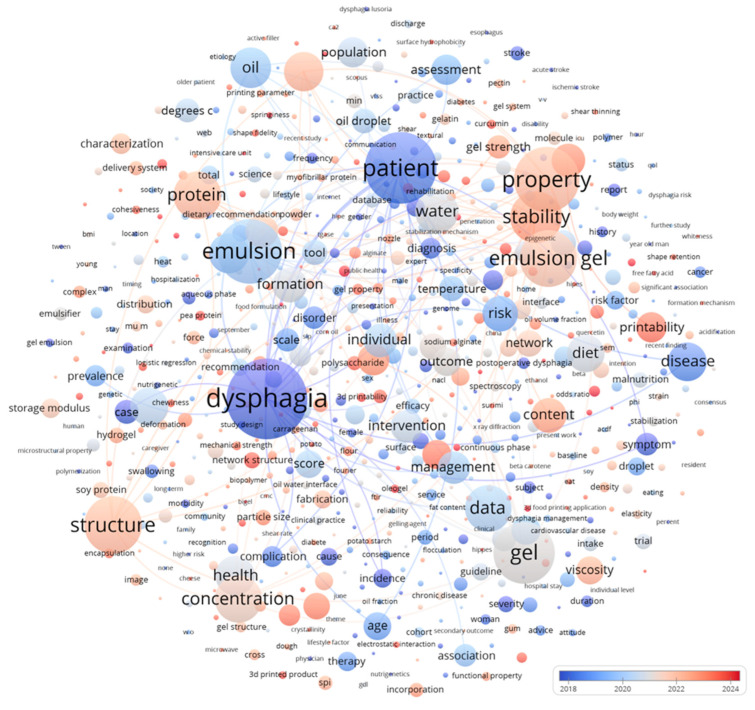
Keyword co-occurrence map of gel-based 3D food printing research for dysphagia and personalized nutrition.

As illustrated in Figure 1, dysphagia-oriented 3D food printing is transitioning from proof-of-shape demonstrations to clinically reliable gel-based platforms [6,7]. Current directions emphasize process-informed gel design, multiphase emulsion gels that couple energy density with oral lubrication, and recovery, fracture, and tribological performance that preserves texture under realistic serving and consumption conditions. Conventional dysphagia diets are typically provided as purees, minced foods, or thickened liquids [8]. Although these formats can improve safety by slowing bolus transit and increasing cohesion, they often produce meals with limited visual appeal, reduced variety, and compromised nutrient density because water addition and intensive mechanical disruption dilute energy and eliminate recognizable structures. This limitation is not merely esthetic. Visual cues and structural heterogeneity are closely linked to perceived freshness, appetite stimulation, and willingness to eat. When texture-modified meals are repeatedly served as homogeneous portions, intake commonly declines. Consistency is also difficult to maintain in clinical and long-term care environments [9]. Foods labeled at the same texture level can differ substantially in hardness, adhesiveness, particle size distribution, and yield behavior, and robust quality control is challenging when texture is produced by manual blending and variable thickener dosing.

Additive manufacturing, particularly extrusion-based three-dimensional food printing, offers a pragmatic route to standardize both geometry and mechanical performance while enabling individualized nutrition [10,11,12]. The most established implementations are variants of direct ink writing, in which a viscoelastic edible ink is deposited layer by layer to construct a three-dimensional architecture [13,14]. This approach provides design freedom that is directly relevant to dysphagia care. First, geometric control supports recognition and dignity; reshaping pureed foods into familiar forms can improve acceptance. Second, the digital workflow enables portion control and nutrition personalization, allowing energy density, protein dose, and micronutrient fortification to be specified by design rather than by estimation [15]. Third, printing supports microstructure programming through multi-material deposition and controlled post-processing. This capability is essential because dysphagia-safe foods must satisfy competing requirements: they should be sufficiently soft to minimize chewing demand yet cohesive enough to resist fragmentation and prevent oral residue. They must yield under low oral stresses while remaining dimensionally stable enough to retain their printed form.

Over the past three years, the field has shifted noticeably in both material selection and performance criteria. Early studies often prioritized achieving a printable paste, frequently through hydrocolloid thickening, and treated shape fidelity as the primary success metric [16]. More recent work increasingly frames the ink as a designed soft-matter system, commonly a gel or gel-like composite, that must perform across the entire printing and consumption chain. Accordingly, research has expanded to the engineering of yield stress, thixotropy, viscoelastic recovery, and interlayer adhesion while also addressing storage stability, reheating behavior, microbial safety, and digestive outcomes. In parallel, dysphagia-focused studies have moved toward objective evaluation frameworks such as the International Dysphagia Diet Standardization Initiative (IDDSI) [17]. The IDDSI provides practical classifications of drink thickness and food texture using simple, repeatable tests, including the syringe flow test for liquids and fork pressure, fork drip, and spoon tilt tests for foods. Embedding these criteria into material design is critical for translation because it bridges the operational language of clinicians with the quantitative descriptors used in food and materials engineering.

This review focuses on gel-based material systems because gels offer a unifying platform for aligning printability, swallowing safety, and nutritional delivery. Food gels can be constructed from polysaccharides, proteins, or hybrid networks and can incorporate dispersed phases such as oil droplets, particles, or air. By tuning network connectivity and phase morphology, key attributes including hardness, adhesiveness, lubrication, and bolus cohesiveness can be systematically modulated. Importantly, gelation can be triggered post-deposition to lock in geometry without excessively increasing viscosity prior to printing. This design feature is well matched to dysphagia-oriented manufacturing constraints.

We seek to bridge food science and materials science perspectives by organizing the literature around structure–process–function relationships. We first review gel-based material platforms and the underlying gelation mechanisms. We then survey additive manufacturing modalities, process control strategies, and emerging approaches to texture programming. Next, we discuss IDDSI-oriented evaluation and mechanistic links to oral processing, with an emphasis on rheology and tribology. We subsequently summarize application case studies relevant to older adults and dysphagia and address the safety, stability, and quality systems required for clinical translation. Finally, we outline key challenges and propose a research agenda that prioritizes reproducibility, digitally enabled manufacturing workflows, and clinically meaningful endpoints.

## 2. Gel Platforms for Dysphagia-Friendly 3D-Printed Foods

### 2.1. Linking Print Physics to Swallowing Mechanics in Soft Solids

Gels are arguably the most versatile soft-matter platform for dysphagia-oriented extrusion because they can be engineered to satisfy two requirements that are often antagonistic [18]: they must transiently liquefy under the high-shear conditions experienced inside the nozzle, yet rapidly rebuild a load-bearing microstructure after deposition. At the point of consumption, they must form a cohesive bolus under saliva dilution, oral shear, and low-force compression because many dysphagia patients exhibit reduced tongue pressure and limited oral control. Accordingly, the gel should provide sufficient yield stress and structural stability to retain its printed form before swallowing while still yielding and breaking down under relatively weak oral forces without requiring strong chewing. Accordingly, gels should not be treated as convenient “thickeners.” A more useful framing is that dysphagia printing constitutes a coupled process chain: manufacturing imposes a defined deformation and thermal history, whereas oral processing imposes a second, evolving mechanical and chemical history that is strongly shaped by dilution, lubrication, and time-dependent structural reorganization. This requirement becomes even more critical during the pharyngeal stage, where delayed swallow initiation or impaired airway protection can increase aspiration risk. Under these conditions, gels should resist brittle fracture and particulate breakup because fragmentation into small particles can compromise bolus integrity and increase the likelihood of airway invasion.

From a swallowing-mechanics perspective, dysphagia should not be treated as a single impairment, because swallowing safety depends on a coordinated sequence of events, including oral containment, bolus formation, oral propulsion, timely laryngeal closure, and efficient pharyngeal clearance [19]. Impairment may occur at multiple stages of this sequence, and dysfunction at different stages places distinct demands on gel-based ink design. During the oral phase, reduced tongue pressure, impaired mastication, poor bolus control, and limited chewing efficiency can compromise bolus consolidation and oral transport [20]. Accordingly, a printable gel should exhibit sufficient yield stress and shape stability to maintain its structure before swallowing while still being easily deformed under relatively low oral forces. During the pharyngeal phase, delayed swallow initiation, weakened airway protection, and inefficient bolus clearance increase the risk of aspiration, particularly when the bolus fragments into small particles or leaves pharyngeal residue. Therefore, dysphagia-oriented gels should not only be soft but also be sufficiently cohesive to resist fragmentation; maintain bolus integrity during shear, compression, and saliva dilution; and support safer swallowing [21]. Accordingly, dysphagia-oriented ink design should be considered not only in terms of printability but also in relation to stage-specific swallowing requirements. A key implication is that yield stress alone is an insufficient design target. Many inks can be formulated to exhibit an apparent yield point yet still print unreliably due to wall slip, filament tearing, or slow structural recovery [22]. Conversely, an ink may print with high fidelity but fail clinically if it becomes tacky, leaves pharyngeal residue, or fragments during oral processing. What ultimately matters is the time-dependent rheological signature and how it maps onto printer-specific conditions, particularly thixotropic breakdown and recovery kinetics at relevant shear rates, nozzle geometries, and layer times. To move beyond printer-specific demonstrations, the field requires protocol-level comparability, including standardized pre-shear conditioning, recovery tests conducted on deposition-relevant timescales, and explicit reporting of slip-corrected data when smooth nozzles or high-water-content systems are used [23].

Gels further enable staged structuring strategies that can decouple extrusion performance from post-deposition stability and downstream handling demands, including chilling, reheating, warm holding, and transport. However, multi-stage structuring is not inherently advantageous for dysphagia applications. Post-print strengthening can elevate fracture risk, reduce lubrication, or generate spatial heterogeneity if triggers propagate unevenly through a printed lattice. The central design challenge is therefore to construct microstructures that remain robust under realistic handling conditions while preserving tolerance to saliva dilution and gentle oral deformation. Meeting this goal requires treating dilution stability, thermal cycling tolerance, and storage-induced aging as primary constraints rather than secondary considerations.

**Figure 2:**
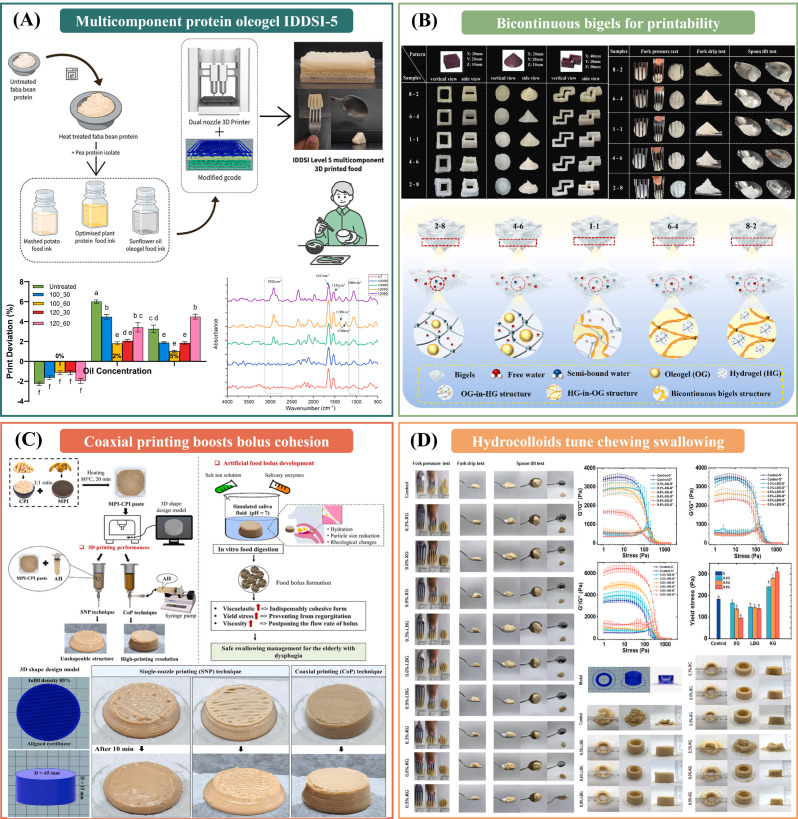
Converging strategies for 3D-printed dysphagia foods. (**A**) Hydrocolloid-tuned mushroom inks link rheology, print fidelity, and bolus behavior [24]. (**B**) Bicontinuous bigels balance shear thinning, recovery, and moisture partitioning for IDDSI compliance [25]. (**C**) Coaxial printing stabilizes fragile protein gels and strengthens saliva-conditioned bolus cohesion [26]. (**D**) Multicomponent designs co-optimize macronutrients, texture, and IDDSI Level 5 performance [27]. (Different letters represent the significance analysis of the data).

The four representative studies in Figure 2 collectively depict a coherent path from ink design to the performance of 3D-printed foods for those with difficulty swallowing. Hydrocolloid-modified mushroom inks show that tuning yield stress, adhesiveness, and texture can improve shape fidelity while shifting chewing time, oral comfort, and bolus rheology. Bigel inks further demonstrate how a bicontinuous oleogel-hydrogel network provides balanced shear thinning and rapid thixotropic recovery, together with stable moisture distributions, thus enabling self-support during printing and IDDSI compliance. Coaxial printing reframes printability as a process-architecture variable, as an alginate sheath can stabilize fragile protein gels and, after saliva interaction, promote a more cohesive bolus with higher viscoelasticity and viscosity. Finally, multicomponent constructs integrate starch bases, plant-based protein inks, and oleogels to co-design nutrition and texture while meeting IDDSI Level 5. A key next step is to standardize saliva dilution, time-scale-matched rheology, and tribology protocols so that results can be compared across printers and laboratories and used to predict clinically relevant outcomes and storage-related texture drift. Furthermore, storage and reheating effects should be incorporated into evaluation workflows because dysphagia foods must remain safe and palatable after processing.

### 2.2. Mechanism-Aware Design for Polysaccharide Networks

Polysaccharide gels dominate food printing because they are broadly food-grade, low-cost, and readily tunable through concentration, ionic environment, and temperature modulation [28]. However, their widespread use has also encouraged template-driven formulations, in which systems are assembled by analogy rather than by mechanism. A more rigorous view recognizes that polysaccharide networks differ not only in nominal gel strength but also in relaxation spectra, failure modes, syneresis tendencies, and sensitivity to ions and thermal history. These differences directly govern printing fidelity and dysphagia performance, particularly under practical conditions that include warm holding, chilling, reheating, and saliva dilution [29].

Ionically crosslinked networks exemplify both opportunity and risk [30]. Calcium-mediated gelation can rapidly immobilize filaments and reduce slump, including in workflows that employ coaxial extrusion or post-print immersion [11]. Yet the same mechanism can generate spatially non-uniform crosslink density because ion diffusion is inherently heterogeneous within printed geometries. Clinically, this heterogeneity is consequential: highly crosslinked regions may fracture into discrete fragments, whereas under-crosslinked regions may collapse or smear, yielding inconsistent textures within a single serving [31]. Over-reliance on rapid ionic setting can also narrow the processing window, making outcomes highly sensitive to the calcium source, release kinetics, and local water activity. Future ionically crosslinked scaffolds should therefore be engineered around controlled ion delivery and bounded crosslink gradients by using strategies such as chelator-mediated buffering, internal ion reservoirs, or spatially programmed crosslinking that avoids abrupt discontinuities.

Pectin provides a contrasting case in which compositional flexibility expands the design space while increasing translational complexity [32]. Low-methoxyl pectin can form calcium-bridged junctions, whereas high-methoxyl pectin gels form these junctions through sugar–acid-mediated associations. This duality is advantageous for fruit-based matrices and reduced-sugar formulations, but it complicates standardization across clinical sites because texture drift can arise when the degree of esterification, acetylation, or molecular-weight distribution varies between batches [33]. For pectin to function as a reliable dysphagia-friendly printing scaffold, reporting should move beyond brand identifiers toward material specifications and functional characterization that predict performance after realistic storage and reheating and not solely immediately after printing.

Thermoreversible gels such as gellan, carrageenan, and agar are well suited to “print-then-set” workflows, but their gelation kinetics and resulting microstructures depend strongly on the cooling rate, ionic conditions, and co-solutes, including sugars and proteins. These dependencies amplify scale-up risk because thermal gradients and residence times differ substantially between laboratory printers and production environments such as hospital kitchens. Agar is particularly instructive: it can form high-strength gels at low concentrations, yet it often fails due to brittle fracture, which is undesirable when cohesive deformation is required for safe swallowing [34]. This does not render agar unsuitable. Rather, it implies that brittleness suppression and energy dissipation should be explicit design objectives, typically addressed through mixed-gel strategies or targeted network modifiers selected to shift the failure mode and relaxation behavior [35].

Blends, including konjac glucomannan combined with synergistic gums, highlight a broader principle: single hydrocolloids rarely optimize print recovery and oral acceptability simultaneously [36]. Mechanistically guided blending can assign distinct functional roles; for example, one component provides yield stress and shear thinning, while a second one accelerates elastic recovery and water binding, and a third one tunes lubrication or suppresses syneresis. A persistent gap is that many blend studies terminate at macroscopic rheology and do not resolve how phase separation, entanglement density, or junction-zone morphology evolve during printing and subsequent storage [37]. Without microstructural resolution, blend performance remains vulnerable to ingredient variability and process drift.

Starch-based gels warrant particular emphasis because they align with sensory familiarity and cost constraints and can be nutritionally preferable to high-gum systems. The central limitation is that starch networks are intrinsically time-dependent. Retrogradation and water redistribution can stiffen printed foods during cold storage, creating non-compliance at the point of consumption rather than at the time of printing [38]. Contemporary strategies increasingly treat starch as an engineerable matrix by incorporating hydrocolloids, lipids, or enzymes to regulate chain reassociation and water mobility. A more ambitious direction is to deliberately broaden the relaxation spectrum so that the system recovers rapidly after extrusion while remaining low in adhesiveness and resistant to brittle fracture [39]. Achieving this outcome will likely require coordinated control of starch granule morphology together with secondary network design, rather than simply increasing the gum concentration [40].

### 2.3. Topology and Failure Control in Protein and Mixed Networks

Protein gels offer higher nutritional density and additional microstructural control variables, but their advantages in 3D food printing are often presented as more straightforward than they are in practice [41]. Protein-based inks commonly exhibit substantial variability arising from source-dependent composition, processing history, and strong sensitivity to pH, ionic strength, and thermal exposure. These sensitivities are not minor formulation details. They are determinative of whether a protein ink can remain robust under the variability typical of clinical preparation, where water quality, heating trajectories, and holding times are difficult to standardize tightly [42].

Heat-set gels derived from whey, egg, soy, and pea can be steered toward fine-stranded or particulate network architectures depending on the denaturation and aggregation pathways [43]. Fine-stranded networks often produce elastic, water-retentive gels that deform cohesively, whereas particulate gels more closely resemble jammed aggregates with distinct yielding, fracture, and recovery behaviors. Many reports target a modulus window and implicitly assume that meeting this criterion confers dysphagia relevance. This assumption is unreliable because swallow-relevant performance is governed not only by small-strain stiffness but also by failure mode, lubrication, and residue formation [44]. A gel may fall within a nominal modulus range yet still perform poorly if it adheres to mucosa, leaves persistent residue, or disintegrates into granules under low-force compression. These considerations motivate integrating rheology with tribology and fracture characterization and treating failure mode as a primary design outcome rather than an incidental observation [45].

Plant proteins are attractive for sustainable fortification and potentially more recognizable textures, but limited solubility and heterogeneous aggregation frequently compromise print repeatability [46]. Physical modification, enzymatic treatment, and polysaccharide complexation can improve their functionality, yet these interventions may also change their flavor, color, and digestibility [47]. From a dysphagia standpoint, the central unresolved challenge is balancing protein enrichment with safe, cohesive bolus formation under saliva dilution. High-protein matrices can become chalky or generate particulate residues during oral processing. Emulsion gel and microgel strategies may mitigate these risks by distributing protein as structured particles and interfacial stabilizers rather than enforcing a continuous, rigid protein network. However, such approaches require validation under realistic oral processing conditions, rather than relying on the texture at rest.

Hybrid protein–polysaccharide gels are frequently described as synergistic, but synergy is conditional and often confined to narrow composition and processing windows. Electrostatic complexes and coacervate-like associations can increase yield stress and suppress phase separation, yet they may be highly sensitive to modest shifts in pH and ionic strength, which commonly occur during preparation, reheating, and saliva exposure. Translation therefore requires engineering hybrids with built-in tolerance, for example by selecting interaction regimes that remain stable across a practical pH band, using buffering strategies compatible with taste and safety, and implementing process controls that constrain thermal history [48]. Mechanistically, a high-value research direction is to map composition to network topology and then link topology to swallow-relevant failure. Proteins can function as load-bearing strands, interfacial stabilizers, or microgel fillers, and each role implies distinct sensitivities to dilution, shear history, and thermal cycling [48].

### 2.4. Interfacial Mechanics in Emulsion Gels and Particle-Stabilized Inks

The transition from single-phase hydrogels to multiphase emulsion gels represents one of the most consequential material advances in dysphagia-oriented 3D food printing because it directly addresses two clinically relevant constraints: increasing energy density without increasing serving volume and tuning lubrication without creating free oil separation. Emulsion gels, high-internal-phase emulsions, and Pickering-stabilized architectures can generate yield stress and elastic recovery through droplet crowding, droplet–droplet interactions, and interfacial network formation, rather than by simply raising the biopolymer concentration. This shift can expand the printability window while reducing perceived stickiness and improving palatability, which often limits acceptance, compliance, and intake [49].

However, it is important to avoid assuming that incorporating droplets inherently improves swallow safety. Increased lubrication is not universally beneficial if it promotes pharyngeal residue or if oil is expressed during oral processing [50]. Accordingly, stability should be evaluated under realistic perturbations, including thermal cycling, shear histories relevant to extrusion and oral manipulation, and dilution conditions representative of saliva exposure, rather than under quiescent storage alone. Pickering stabilization using protein- or polysaccharide-based particles can enhance resistance to coalescence, yet particle identity and mechanics strongly influence mouthfeel. Rigid particles may introduce graininess and persistent residues, whereas soft, deformable microgel particles can preserve creaminess while maintaining interfacial integrity [51]. For dysphagia inks, this motivates a design principle that prioritizes compliant interfacial particles with controlled size distributions, together with friction-relevant measurements that explicitly connect interfacial mechanics to perceived lubrication.

A persistent research gap is the limited mechanistic linkage between microstructures, process performance, and swallow-relevant function. Predictive frameworks that couple droplet size distributions, interfacial rheology, and bulk recovery kinetics to print fidelity and bolus cohesion remain underdeveloped. Many studies report droplet size metrics and bulk moduli as largely independent descriptors. Future work should quantify interfacial viscoelasticity and explicitly relate it to filament fusion, interlayer adhesion, and low-force fracture behavior using standardized in vitro oral processing protocols that incorporate saliva analog dilution and tribological testing [52]. Without such structure–process–function linkages, emulsion gels risk remaining as compelling demonstrations rather than reliable, clinically deployable platforms.

The studies shown in Table 1 demonstrate three dominant routes to “fat without free oil” in dysphagia-oriented printing. HIPE packing with biopolymer-reinforced interfaces include Pickering networks built from protein or protein–polysaccharide particles. Emulsion-filled gels containing droplets act as soft fillers that tune yield and recovery while lowering perceived stickiness. The recurring limitation is that most papers rely on bulk rheology and static microstructures, while swallow safety depends on what happens during dilution, lubrication, and gentle oral shear. Only a few studies begin to include friction-related measurements or clinically framed IDDSI outcomes [49,53]. A strong next step is to standardize in vitro oral processing workflows that combine saliva analog dilution, tribology, and low-strain fracture tests, then link those outputs to interfacial viscoelasticity and droplet or particle size distributions. This would turn interfacial mechanics from a descriptive feature into a predictive design variable for print fidelity, bolus cohesion, and residue risk.

### 2.5. Phase Continuity and Reheating Realism in Bigels

Bigels integrate an oleogel with a hydrogel to form a biphasic, structured soft material [61]. Their primary appeal is the ability to incorporate structured lipids without releasing free oil while retaining a compliant aqueous phase that supports cohesive deformation. In principle, bigels can increase energy density, tune lubrication, and enable delivery of lipophilic nutrients. In practice, their stability challenges are frequently underestimated in printing contexts because phase continuity and interfacial adhesion determine whether the construct behaves as an integrated soft solid or as a weak composite that undergoes separation under shear or during storage [62].

From a manufacturing perspective, bigels can be highly shear-sensitive [63]. Extrusion may disrupt bicontinuous morphologies, shift domain-size distributions, and impose anisotropy that later appears as a direction-dependent fracture or spatially uneven mouthfeel. From a dysphagia perspective, lipid-rich surfaces may reduce friction, yet any tendency toward oil exudation represents a safety and acceptability concern, particularly under warming and holding conditions that are common in clinical workflows [64]. Bigel design should therefore prioritize interfacial stabilization as an explicit objective by using strategies such as particle-reinforced interfaces, protein-based compatibilizers, and mixing protocols that reproducibly set the domain size and connectivity. Additionally, performance should be validated after reheating and warm holding because phase migration and interfacial weakening are often latent failure modes that emerge under these conditions rather than immediately after printing.

Looking forward, bigels can be treated as programmable microstructures rather than as static biphasic mixtures. By controlling the phase ratio and domain geometry through processing, it may be possible to decouple macroscopic softness from lubrication and energy density in ways that single-phase gels cannot readily achieve. Realizing this potential will require microstructural characterization and process mapping that connect morphology to print behavior, thermal-history tolerance, and swallow-relevant failure, instead of relying solely on endpoint texture metrics.

### 2.6. Self-Healing Rheology and Multi-Objective Design in Microgel Inks

Granular gels composed of jammed microgels offer a powerful approach to self-healing yield-stress fluids, making them ideal candidates for dysphagia-oriented 3D printing. These systems can print with high shape fidelity, support complex geometries, and recover rapidly after extrusion. For dysphagia applications, microgel-based inks provide a compelling solution because bulk yield stress can ensure printability while maintaining a creamy mouthfeel if the microgels are soft and lubricious. This ability to balance print stability with oral comfort addresses a long-standing challenge faced by many hydrocolloid-thickened systems.

However, granular systems are highly history-dependent. The packing and interparticle friction of microgels can shift under shear, leading to batch-to-batch variability in extrusion pressure, filament smoothness, and interlayer bonding. Furthermore, their stability under storage and thermal cycling remains uncertain, particularly for protein-based microgels, which may aggregate or expel water over time. To ensure successful clinical adoption, microgel inks must be evaluated under realistic handling conditions, including repeated shear from mixing, pumping, re-extrusion, and warm holding. Additionally, microbial safety must be considered, especially for high-moisture foods prone to contamination.

A key research frontier lies in treating microgels as modular building blocks with tunable stiffness, surface chemistry, and digestion behavior. This opens avenues for protecting sensitive nutrients, controlling enzymatic access, and programming breakdown profiles. In dysphagia applications, breakdown behavior cannot be inferred solely from mechanical stability. Delayed breakdown may improve bolus cohesion, but it can also increase residue formation and reduce bolus clearance. Therefore, integrating oral processing analog tests and digestion kinetics into material design is essential for ensuring that microgels can become clinically meaningful platforms.

In addition to self-healing properties, the field must move toward multi-objective optimization that addresses the complex needs of dysphagia patients. Formulation is not merely an esthetic optimization but a critical part of the diet, especially for individuals with frailty, sarcopenia, and micronutrient deficiencies. The material platform must prioritize nutrition-first objectives while complying with texture standards and remaining acceptable for consumption. This is challenging because fortification often disrupts the gel microstructure. For example, protein enrichment can increase firmness and alter fracture behavior, while fiber addition can increase viscosity and adhesiveness, and lipid inclusion can raise energy density but may destabilize networks or create exudation risks if not integrated structurally.

The field would benefit from treating printability, IDDSI-relevant texture outcomes, lubrication comfort, and nutritional targets as simultaneous constraints in a multi-objective design framework. This calls for design-of-experiment strategies, data-driven modeling, and ingredient modularity. A robust scaffold can provide consistent rheology and recovery, while nutrient modules—such as structured emulsified lipids, protein microgels, and encapsulated micronutrients—can be incorporated with minimal disruption to the microstructure. This platform-based mindset is more likely to be transferable across clinical settings than bespoke formulations optimized for specific printers or kitchens.

Progress also hinges on redefining compliance testing. While IDDSI tests are essential for clinical communication, they are relatively coarse and fail to capture the complexities of oral processing, particularly time-dependent changes induced by storage, reheating, and saliva dilution. A more comprehensive framework involves pairing IDDSI classifications with quantitative rheology, fracture, and tribology metrics and validating these metrics against patient-centered outcomes such as residue, comfort, and intake under point-of-service conditions. Without this validation, innovations in materials risk optimizing proxies rather than clinically relevant performance.

In summary, gel platforms have advanced from simply enabling extrusion to facilitating the design of engineered soft foods with programmable microstructures. The next step is to make this engineering clinically robust, which can be achieved by standardizing protocols, accounting for processing and storage histories, and establishing mechanistic links between the microstructure and swallow-relevant outcomes. This shift also supports a transition from one-off ink formulations to platform technologies that can accommodate personalization while ensuring reproducibility in real clinical manufacturing environments.

## 3. Additive Manufacturing, Process Control, and Texture Programming

### 3.1. Extrusion Deposition as a Repeatable Process

Extrusion remains the dominant method for dysphagia-oriented food printing due to its ability to deposit viscoelastic, high-moisture formulations across a wide range of compositions using relatively simple hardware. However, a common misconception is that extrusion simply translates rheology into geometry. Extrusion is a high-intensity process that continuously alters the material state through shear, extensional flow, pressure-driven compaction, residence time, and wall interactions. For gel-based inks and precursors, these processes can rearrange network junctions, break weak associations, induce phase migration, and create anisotropy in the filament microstructure. These changes are not merely cosmetic; they can lead to failure modes such as tearing along deposition lines, brittle fracture at weak interlayer interfaces, or smearing and residue formation when shear-induced structure loss causes excessive surface adhesiveness [65]. Therefore, a review focused on clinical translation should treat deposition as a controllable unit operation, with measurable inputs, state variables, and outcomes, rather than as a neutral delivery mechanism.

Reproducibility issues are currently more related to the lack of defined deposition boundary conditions than to the absence of printable materials. Many studies report on nozzle diameter and print speed, assuming comparability, but the actual dose delivered per unit path length depends on factors such as volumetric flow accuracy, pressure dynamics, and the mechanical compliance of the extrusion system, where factors including tubing elasticity, trapped air, and temperature-dependent viscosity change inside the cartridge. Even when a printer maintains a constant speed, pulsatile flow can occur due to stepper microstepping, pressure relief events, or partial clogging, all of which can cause periodic changes in filament diameter and intermittent layer fusion loss [66]. Wall slip, another often underreported variable, decouples bulk rheology from apparent extrusion behavior, particularly in high-water-content gels, smooth nozzles, and formulations containing lubricating dispersed phases [67]. When slip is dominant, the process becomes sensitive to nozzle surface finish, minor compositional changes, and pre-shear history, meaning a formulation that “prints well” in one setup may fail in another, even with similar nominal settings.

A manufacturing-centered approach reframes extrusion printing around process signatures and capability, rather than isolated best-case prints [68]. Future research should focus on quantifying the time-resolved pressure and flow response during representative toolpaths and linking these signals to filament geometry, interlayer weld strength, and the final texture under realistic serving conditions. A useful concept is to describe the operating window not as a narrow region where collapse is avoided, but as a broader range where key outcomes show low variance despite realistic disturbances, such as ingredient variability, small temperature fluctuations, and brief pauses in printing. This is particularly crucial in healthcare settings, where consistency is often more important than peak resolution. Progress will likely stem from standardizing a minimal deposition metrology package suitable for food-safe environments, including pressure sensing near the nozzle, motor current proxies for extrusion resistance, gravimetric flow verification, and in-process filament width stability measurement. If these metrics are reported in a consistent manner, they can provide the foundation for cross-laboratory transfer and transform today’s largely descriptive “printability windows” into more portable, process-defined design spaces.

**Figure 3:**
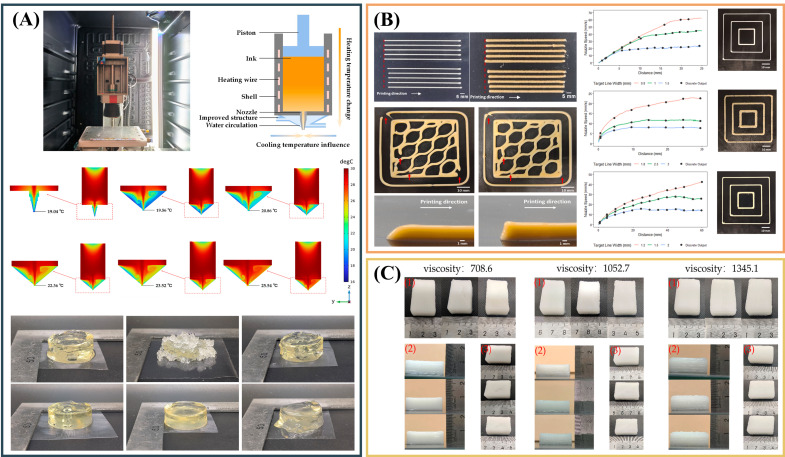
Process-defined extrusion control in food 3D printing. (**A**) Nozzle temperature windows govern ink state transitions and line stability [70]. (**B**) Computer vision sensing of the extrusion rate and filament width enables feedforward motion control (The red arrows indicate the pressurizing positions of the printing path) [69]. (**C**) Collaborative temperature–pressure dual closed-loop control suppresses pressure fluctuation and improves dimensional accuracy ((1), (2), and (3) represent different sample observation angles.) [65].

The three studies in Figure 3 reposition extrusion printing as a measurable unit operation rather than a simple rheology-to-shape translation. Nozzle temperature control reveals sharp state transitions in low-viscosity inks, where a narrow thermal window switches deposition from grainy to stable lines, while overheating triggers flow and collapse. Vision-based monitoring converts extrusion into a quantified signal, enabling feedforward nozzle-motion control that corrects the line width and suppresses under-extrusion across diverse foods. Finally, dual closed-loop control of temperature and pressure stabilizes forming accuracy by reducing pressure fluctuations and compensating for rheology drift under external disturbances [69]. Next, portable process signatures should be standardized as a common language between materials and machines. If pressure or extrusion stability patterns can be compared across printers, they can explain why the same ink succeeds in one setup but fails in another. Linking these signatures to interlayer bonding and the final serving texture would also shift the field from “good-looking prints” to reliability and safety. Most importantly, the goal should be robustness. Designs that keep performance and not just peak accuracy stable under small disturbances in ideal runs are encouraged.

### 3.2. Thermal History and Setting Kinetics as Coupled Control Variables

Temperature is often viewed as a practical constraint in gel-based food inks, but it is more accurately treated as a control variable that influences structure formation, layer fusion, and time-dependent texture changes. A single cartridge or bed setpoint is rarely sufficient, as the relevant factor is the thermal trajectory experienced by the ink. During printing, the material may warm during holding, undergo shear heating during extrusion, cool while traveling through the nozzle and ambient air, and then experience variable post-deposition heat transfer, which depends on factors such as geometry, layer time, and the thermal properties of the underlying substrate [71]. These time-varying thermal paths determine whether filaments fuse into a continuous phase or remain weakly bonded, whether slumping occurs before setting, and whether stiffness gradients develop across thick constructs. In dysphagia applications, these effects are particularly critical, as the target textures often lie near threshold values where even minor changes can move a product from acceptable to unsafe or undesirable [72].

Thermal control also interacts with oral processing in ways that are frequently overlooked. A construct that is dimensionally stable may still fail clinically if its thermal history results in a brittle network with low fracture energy or if surface dehydration leads to increased tackiness and residue formation. Additionally, dysphagia workflows often involve chilling, storage, transport, and reheating, meaning that the thermal trajectory during printing is only one part of the overall thermal cycle. Gel networks can continue to evolve during holding through retrogradation, further junction formation, water migration, or phase separation [73,74]. This indicates that the performance target should not be the texture immediately after printing but rather the texture at the time of service, which can be achieved after considering the thermal and storage processes it undergoes in practice.

A more research-driven approach would be to couple thermal management with simplified thermal–structure models that predict setting kinetics and texture changes over time. These models do not need to be highly detailed to be useful [69]. Even reduced-order models that link temperature trajectories to gelation rate, network formation, and interlayer bonding can guide robust parameter selection and help avoid fragile recipes. Closed-loop strategies become particularly valuable, not only for maintaining a temperature setpoint but also for controlling a time-dependent thermal profile that optimizes both buildability and the final swallowing mechanics. This could involve adaptive bed heating for consistent interlayer bonding, controlled cooling rates to prevent brittle microstructures in thermoreversible gels, and thermal limits that protect heat-sensitive nutrients and aromas while still achieving proper setting. A translationally aligned thermal strategy should treat buildability, nutritional retention, and serving-time texture as simultaneous constraints, and it should evaluate constructs after realistic holding and reheating to reveal any drift that might otherwise be overlooked.

The studies presented in Table 2 highlight that thermal history and setting kinetics are critical control variables in designing edible gel inks for 3D printing. Temperature trajectories during and after printing influence gelation pathways, network strength, and interlayer bonding, which subsequently affect shape retention, mechanical integrity, and texture changes over time. For instance, cooling and differential scanning calorimetry (DSC) measurements demonstrate how gel transition points determine the final elastic modulus and layer adhesion, while freeze–thaw cycling experiments show how thermal exposure impacts elasticity and structural stability. Rheological models based on oscillatory temperature ramps further emphasize that thermal preparation dictates flow and recovery behaviors, which are essential for printing precision. However, existing studies often focus on thermal effects at discrete points rather than on continuous temperature trajectories, which limits the ability to predict post-print performance. Additionally, the integration of in situ thermal sensing with setting kinetics and microstructural evolution remains underexplored. Future research should therefore focus on developing closed-loop thermal management systems combined with reduced-order kinetic models that can predict gelation, interlayer bonding, and texture drift throughout the entire printing and service process. This approach will ultimately enable the creation of robust design spaces for safe, dysphagia-compatible printed foods.

### 3.3. Triggered Curing and Heterogeneity Management in Printed Geometries

Triggered curing strategies, such as ionic crosslinking, pH-driven setting, and enzymatic bridging, are commonly used to maintain ink extrudability during deposition while enhancing strength afterward [79]. The primary translational challenge is that most triggering methods are diffusion-limited in printed geometries, making heterogeneity a predictable outcome unless actively managed. As curing triggers penetrate a filament or lattice, gradients in crosslink density or reaction extent can lead to the formation of stiff skins, weak cores, or localized hard domains. These internal variations may not be visible through external geometric inspection but can significantly affect oral failure behavior, leading to fragmentation, delamination, or persistent residue. In dysphagia applications, this is particularly critical because safety relies on cohesive deformation under gentle loads, and even small amounts of brittle regions can cause unacceptable particle formation [80].

Much of the current research focuses on rapid curing to enable the creation of tall structures and fine features [81]. However, for dysphagia-oriented foods, the key objective is to achieve bounded and uniform strengthening that preserves ductile deformation and cohesive bolus formation under dilution. This reframes curing from a problem of speed to one of uniformity and tolerance. Rate control becomes valuable, not because faster curing is inherently better, but because controlled kinetics can prevent sharp gradients and reduce sensitivity to small variations in environmental conditions. The distribution of curing triggers becomes central, particularly in how they are introduced, how they partition in multiphase systems, and how factors like water activity and temperature during holding influence their effectiveness. Microstructural uniformity should also be assessed in geometries that more closely resemble real products, as diffusion lengths and surface-to-volume ratios vary dramatically across lattices, thick sections, and multi-material interfaces.

Hybrid curing strategies offer a promising solution to minimize gradients [82]. By combining a mild, distributed pre-structuring mechanism with a slower, more uniform secondary strengthening process, it is possible to reduce heterogeneity and improve robustness. For example, a weak physical network can stabilize the shape immediately, while a controlled chemical or ionic process develops strength more uniformly over time. Similarly, internal triggering mechanisms that generate crosslinking agents within the bulk material can reduce surface-driven gradients compared to external baths, provided that their kinetics are carefully controlled. Importantly, validation should move beyond static modulus targets measured immediately after curing [44]. For dysphagia applications, curing strategies should be evaluated based on the failure mode under saliva-like dilution and combined shear–compression and on their ability to maintain cohesive deformation after post-processing steps, such as reheating. Without these outcome-aligned tests, curing innovations risk optimizing structural stiffness at the cost of swallow reliability.

### 3.4. Monitoring, Feedback Control, and Predictive Models for Clinical Reliability

Healthcare-oriented deployment highlights a gap between feasibility and reliability. Many studies demonstrate that formulations can be successfully printed once under controlled conditions [83,84,85], but clinical translation requires consistent printing across operators, sites, and days, with serving-time texture remaining within defined bounds. This is fundamentally a control problem, meaning that monitoring and feedback are essential components rather than optional add-ons. While vision-based tracking of filament width and layer placement is useful for directly addressing geometry, it is incomplete because it cannot reliably detect internal defects such as poor layer fusion, voids, or curing gradients. Food printing also imposes unique constraints compared to other additive manufacturing fields. Sensors must tolerate fouling, withstand washdown, and remain food-safe, limiting approaches that rely on exposed optics, fragile contact probes, or complex in-nozzle instrumentation.

A practical near-term solution is to integrate multiple low-burden signals into a reliability framework. Pressure sensors near the nozzle can capture clogging onset, slip regime shifts, and pulsatile flow. The extruder motor current can act as a proxy for resistance changes that precede visible defects. Thermal sensing can detect drift affecting setting kinetics and interlayer bonding, while limited vision can verify external geometry and detect gross placement errors. Together, these signals can enable closed-loop adjustments, such as flow compensation during viscosity drift, pause handling with controlled re-priming, or temperature corrections during long prints. The key is not to pursue maximal sensing, but to capture the minimum set of signals that explain most of the variance in the outcomes critical for serving-time texture and swallow safety.

Predictive models and digital twins are frequently proposed to reduce trial and error in this process. Their true value lies in standardization across equipment and sites by mapping measurable inputs, such as rheology, thermal properties, and flow-control behavior, to clinically meaningful outcomes. This requires more reliable targets than subjective printability scores. Datasets should include endpoints that reflect time-dependent textures, reheating effects, and swallowing-relevant proxies like cohesion under dilution and friction-related residue risks. Additionally, models should be evaluated based on their ability to generalize across different printers and ingredient lots, rather than only within a single laboratory setup. If models are trained on narrow, convenience-based labels, they will optimize surrogates that fail to transfer [86]. A rigorous approach involves defining shared reporting standards for process signals, establishing outcome metrics at serving conditions, and using modeling to identify robust operating windows rather than focusing on single optimal points. In this framework, modeling becomes a tool for risk reduction and consistency assurance, aligning more closely with the needs of clinical nutrition than with the typical goals of maximizing geometric complexity.

### 3.5. Geometry, Multi-Material Architecture, and Post-Processing Robustness in Texture Programming

Additive manufacturing allows for texture programming through both geometry and formulation, a capability that is particularly relevant for dysphagia, where swallowing performance depends on controlled deformation and minimal fragmentation rather than simply low hardness. Infill patterns, porosity, wall thickness, and orientation can influence apparent compliance, collapse behavior, and stress distribution under load. However, geometry-based designs are often validated solely through uniaxial compression, which is a limited proxy for oral processing [87]. In the mouth and pharynx, deformation occurs through a combination of shear and compression under lubrication conditions that evolve as saliva dilutes the bolus. A lattice that appears soft in compression may still tear under shear if stress concentrates at interfaces, and highly porous structures can trap material, increasing residue even if bulk hardness is low [88]. Therefore, geometry-driven designs should be evaluated using deformation modes and dilution conditions that better replicate oral handling, with a focus on failure modes and not just peak force [89].

Multi-material and core–shell constructs expand geometry into spatially patterned mechanics and nutrition but introduce a new primary failure pathway. Differences in setting kinetics, water migration, and bonding can result in weak interphases that delaminate during gentle oral deformation, generating fragments and increasing residue. Robust interface design should be viewed as an engineered interphase, rather than an assumed outcome of co-printing [90]. This involves matching rheological recovery to ensure adjacent materials weld effectively, tuning curing schedules to prevent one phase from locking before the other can fuse, and controlling water activity gradients that lead to interfacial weakening during storage. From a research perspective, interfacial strength and fracture should become routine measurements for multi-material dysphagia foods, as they often govern clinical failure more than the bulk properties of the individual phases [24].

Post-processing adds another layer of complexity, as printed foods are typically chilled, stored, transported, and reheated. Network aging, water migration, syneresis, and multiphase instabilities can alter texture after printing, sometimes enough to surpass safety-relevant thresholds. This highlights the need for integrated design, where geometry, curing strategies, and post-processing protocols are optimized together, and performance is evaluated at serving conditions and not just at the time of printing. This approach also calls for a shift in how texture programming is conceptualized. The goal is not to encode a single mechanical state into the printed object but to encode a trajectory of acceptable mechanical states across the handling chain, with predictable failure modes that remain cohesive under dilution and gentle loads [89]. Achieving this level of robustness would elevate dysphagia food printing from impressive demonstrations to reliable clinical manufacturing and provide a clearer scientific foundation for translating texture programming into measurable improvements in safety, comfort, and intake.

## 4. IDDSI-Oriented Evaluation and Mechanistic Links to Oral Processing in Gel-Based Printed Foods

### 4.1. IDDSI as a Translational Scaffold for Gel Platforms

For gel-based dysphagia foods, the IDDSI framework serves as a practical and effective translational tool, converting clinically meaningful but often implicit texture judgments into deployable, low-cost checks that can be applied across various care settings [91,92]. This practicality is precisely why the IDDSI has become the key interface between material design and dysphagia practice [93]. However, the very design philosophy that gives the IDDSI its practicality also makes it intentionally low-resolution. While it categorizes foods in ways that are robust to routine variability, it does not address the mechanistic differences that can separate a gel that is simply compliant from one that is reliably swallowable under real oral conditions. In gel printing, this gap becomes more evident because additive manufacturing broadens the design space not only in formulation but also in process history and spatial architecture. Two constructs can meet the same IDDSI level but still exhibit different yield mechanisms, recovery kinetics after shear, fracture pathways, and lubrication regimes at mucosal interfaces [25]. These differences are not just theoretical; they are the mechanisms that influence residue formation, perceived effort, fatigue, and bolus consistency when considering factors like saliva dilution and thermal history [94].

A gel-focused perspective also clarifies why the IDDSI alone cannot certify equivalence for printed foods. Gels are characterized by their networked microstructure, which stores and dissipates elastic energy through time-dependent relaxation [95]. Printing introduces directional alignment, layer seams, and potentially gradients caused by thermal trajectories or diffusion-limited curing. While these features may not be captured by spoon tilt or fork pressure tests, they can significantly influence failure during oral handling since oral deformation involves multi-axial and highly localized stresses [96]. A printed gel may pass an IDDSI test but still contain brittle shells from surface dehydration, stiff domains from non-uniform ion diffusion, or weak interlayer welds that become sites for crack initiation under gentle shear.

The most constructive way to position the IDDSI in a gel-centric review is not as a final endpoint, but as a boundary condition. It defines a clinically interpretable acceptance region. The scientific challenge is to identify which gel-state variables, measured under standardized conditions, predict whether a printed product will remain within this region at the time of service. This approach aligns with manufacturing logic: quality cannot be guaranteed by a single pass/fail test if the process may introduce hidden heterogeneity. It must be assured by controlling the gel microstructure and its evolution throughout the entire preparation chain.

In the representative studies presented in Table 3, the IDDSI is consistently treated as a practical clinical interface, while researchers rely on rheology, texture profiling, and structure observations to explain why “IDDSI passing” can still hide meaningful differences in yield, recovery, fracture, and perceived effort. The shared limitation is that most workflows do not standardize process history tightly enough to expose printing-induced heterogeneity, such as interlayer seams, directional alignment, surface dehydration, or diffusion-limited curing gradients, even though these features can dominate localized oral failure. A clear next step involves gel-centric quality control logic, where the IDDSI remains the minimum compliance gate, while release decisions are supported by a small mechanistic panel measured under defined histories, for example, yield plus recovery, fracture or cohesiveness proxies, and lubrication-relevant measures after controlled saliva dilution and thermal cycling.

Clinically, an IDDSI level should be interpreted not merely as a texture label but as a risk management category linked to specific swallowing impairments. For example, IDDSI Level 4 (Pureed/Extremely Thick) is generally suitable for patients with poor tongue control, limited chewing ability, or reduced oral bolus control because the food should hold its shape on a spoon, remain homogeneous, and avoid separation into liquid and solid phases. For gel-based printed foods, this means that IDDSI compliance should be evaluated together with structural homogeneity, resistance to phase separation, and non-fragmenting bolus formation under serving conditions. When IDDSI Level 5 is targeted, the design should additionally ensure that the structure can be broken down by relatively low tongue pressure without generating small, hard particles.

### 4.2. Quantitative Anchors That Translate IDDSI Levels into Gel-State Variables

A central research challenge is mapping categorical IDDSI outcomes to quantitative anchors that are both mechanistically meaningful for gels and operationally useful for process control. In theory, this appears to be a straightforward calibration issue. In practice, however, it is complicated by the fact that gels are history-dependent materials, and printed foods represent an integrated process–structure–property state, rather than simply a formulation-based property. The same gel composition can exhibit different mechanical responses depending on pre-shear conditioning, residence time, thermal trajectory, printing-induced alignment, and post-print curing gradients. Therefore, the mapping cannot be simplified to IDDSI level equals a modulus range. Instead, it should be understood as the “IDDSI level corresponds to a region in gel-state space at the point of consumption,” where the gel-state space includes time-dependent rheology, fracture responses, and interfacial lubrication under dilution [100]. Clinically, rheology matters because it influences bolus transit velocity and the timing of airway protection. A bolus with appropriate viscosity, yield behavior, and elastic recovery can slow premature flow toward the airway, thereby allowing more time for laryngeal closure while still remaining deformable under reduced oral forces [44].

For gel-based platforms, the most transferable anchors are those that account for both structure and kinetics. Yield stress remains valuable when operationally defined with standardized protocols, but it must be interpreted alongside thixotropic breakdown and recovery. This is clinically relevant during the oral phase, where reduced tongue pressure and impaired bolus control mean that a printed gel should neither collapse too easily before propulsion nor resist breakdown to the point of requiring excessive oral effort. A gel that quickly rebuilds its structure after shear can maintain bolus cohesion during oral manipulation, even if its steady-state viscosity is modest. Small-amplitude oscillatory moduli can provide a structural baseline, but their utility increases when combined with time sweeps and recovery tests that replicate layer times and oral processing timescales. In other words, gels should be characterized not only by their stiffness but by how quickly they regain stiffness after deformation and how that regained structure changes under dilution [101]. For 3D printing, these kinetics directly influence interlayer welding and the formation of an effectively continuous gel phase across deposited strands.

Instrumental texture tests, such as penetration or texture profile analysis, often serve as practical proxies, but a gel-focused critique is warranted. Many of these tests emphasize peak force, while swallow-relevant outcomes are typically governed by failure mode and interfacial behavior. A gel with acceptable hardness may still be prone to residue formation if it exhibits high tack or adhesive fracture against compliant surfaces. Conversely, a gel may be soft yet fragment if it has low fracture energy or contains curing gradients [102]. For gel-based printed foods, a more informative approach is to combine shear rheology with compression protocols that simulate oral squeeze flow conditions, then interpret the results through bolus formation logic. This includes evaluating whether the gel deforms cohesively, whether it smears, whether it fractures into discrete pieces, and how these responses change after controlled dilution and thermal cycling.

To ensure field-wide comparability, quantitative anchors should be linked to explicit decisions. The literature would benefit from reporting templates in which each metric is connected to the failure mode it addresses, such as slump during printing, delamination risk between layers, fragmentation tendency under shear–compression, or residue risk under mucosal sliding. In gel terms, this means reporting not just endpoint measurements but also the conditions under which a gel transitions between regimes. These include the stress or strain scale for yielding, the recovery time constant after shear, and the sensitivity of these parameters to temperature and dilution. With this structure, the IDDSI becomes the clinical label, while gel-state variables function as engineering controls [100].

### 4.3. Beyond Shear: Fracture, Extension, and Mucosal Tribology as Gel-Relevant Mechanisms

Oral processing and swallowing are not governed by shear flow alone, and this is where gel platforms either succeed or fail. During bolus manipulation, gels undergo extensional deformation, tearing, and interfacial sliding against mucosal tissues under mixed lubrication conditions [103]. A gel can meet an IDDSI category and still fail if it elongates, strings, and breaks into fragments under extension or if it adheres and coats a surface, leaving residue that complicates bolus clearance [104]. From a pharyngeal safety perspective, this is not merely a mechanical concern because fragmentation reduces bolus cohesiveness and may increase aspiration risk when airway protection is delayed or incomplete. These behaviors are controlled by network topology, the relaxation spectrum, and surface interactions—factors that are not captured by single-point viscosity or a single oscillatory modulus measurement [105]. Tribology complements rheology by describing interfacial sliding between the bolus and oral or pharyngeal surfaces. This is particularly relevant in older adults with xerostomia, for whom even mechanically compliant foods may adhere to mucosal surfaces and leave residue. Gel systems that retain water or release a controlled lubricating phase may reduce friction and swallowing effort, provided that lubrication does not compromise bolus cohesion.

Extensional rheology is especially important for gels that exhibit filamenting or stringiness, such as mixed gels with long relaxation times or multiphase gels where droplets and interfaces contribute to elastic recoil [106]. Capillary breakup or filament stretching tests can determine whether a gel strain hardens, whether it forms long-lived elastic threads, and how quickly it relaxes under extension. These responses can be interpreted mechanistically in terms of network junction lifetime, entanglement density, and the presence of lubricating phases that affect extensional viscosity. For dysphagia applications, the goal is not to eliminate extensibility entirely, as some degree of extensibility can help preserve cohesion. Rather, the objective is to avoid brittle snap and the formation of persistent strings that complicate bolus control [107]. This balance is best approached as a gel design challenge rather than a mere empirical artifact.

Fracture mechanics are equally crucial because the clinical question is rarely “how strong is the gel?” but rather “how does it fail?” Ductile tearing that preserves a cohesive bolus mass is fundamentally different from brittle fracture, which generates discrete fragments that are more difficult to control and may elevate aspiration risk. For printed gels, fracture is often anisotropic because deposition creates oriented filaments and interlayer interfaces that can act as preferred crack paths. Therefore, orientation-aware fracture testing becomes essential, including measurements of tearing resistance or fracture energy along and across the print direction. This is where curing gradients also play a role: a stiff skin over a softer core can create stress concentrations and crack initiation sites, even if average texture metrics appear acceptable.

Tribology links gel design to the interface-dominated failures that lead to residue and perceived effort. Many swallow-relevant outcomes depend on friction between the bolus and mucosal surfaces under low loads and sliding conditions, where lubrication transitions from a boundary to mixed regimes as saliva dilutes and redistributes. The gel microstructure governs this behavior, as water binding, surface hydration, and the presence of free or structured lubricating phases control the formation of a lubricating film. A gel may feel slippery yet leave residue if it forms a persistent coating, or it may feel cohesive yet be difficult to clear if it is too adhesive. A key takeaway for a gel-focused review is that lubrication and cohesion are coupled, not independent. Increasing lubricity through added oil or reduced polymer concentration can lower friction while compromising cohesive integrity under dilution. Similarly, increasing cohesion through stronger networks can not only enhance bolus integrity but also increase tackiness and residue. Multiphase gel platforms, such as emulsion gels and microgel-based granular gels, hold promise because they offer the potential to tune lubrication and yield behavior with partially decoupled knobs, but this is only achievable if interfacial mechanics and particle softness are designed with oral perception in mind. Establishing standardized mucosal tribology protocols and shared gel reference materials would likely accelerate the translation of these technologies more so than additional isolated formulation studies.

### 4.4. In Vitro Oral Processing as a Mechanistic Bridge and a Closed-Loop Design Tool for Gels

Clinical evaluation is essential but not scalable as the primary screening tool for gel formulation and process optimization [108]. In vitro oral processing models can fill this gap if their role is appropriately framed. Their value is comparative and mechanistic rather than definitive. For gel-based printed foods, the most useful in vitro models are those that preserve the coupled features defining oral handling—namely, combined compression and shear, evolving lubrication due to saliva-like dilution, and time-temperature histories that reflect holding and reheating conditions [109]. Without these elements, in vitro tests may overestimate performance by evaluating a gel in an unrealistically static or undiluted state.

A gel-centric in vitro framework should explicitly incorporate dilution kinetics as a key variable. Saliva does not merely reduce viscosity [110]; it alters ionic strength, changes pH locally, introduces enzymes, and redistributes water at interfaces. These factors can weaken physical junctions, destabilize electrostatic complexes, or modify lubrication by altering surface hydration layers. For printed gels, dilution also interacts with the internal architecture [111,112,113]. Porous geometries can accelerate penetration and create heterogeneous softening, while denser regions may retain their structure longer. These dynamics influence bolus cohesion and clearance. In vitro systems that quantify how a gel transitions from a structured solid to a swallowable bolus under controlled dilution would provide a mechanistic link between gel design and clinical risk.

The distinctive advantage of additive manufacturing is that it naturally supports iterative, closed-loop design. Geometry, process parameters, and gel formulation can be updated based on measured outcomes. A credible closed-loop approach would involve printing candidate designs, subjecting them to standardized oral processing analog tests, and then refining design rules using either mechanistic models or data-driven surrogates. For gels, key outcomes should be defined in swallow-relevant terms that can be reproducibly measured, such as cohesive mass formation after dilution, controlled breakup without discrete fragments, low friction without persistent coating, and robustness after reheating. These are not merely sensory claims; they can be operationalized as measurable proxies through combined compression–shear tests, extensional breakup metrics, and mucosal tribology that are all performed after realistic thermal histories.

Modeling can play a role here, but only if it is grounded in gel physics. Reduced-order models that treat the bolus as a time-evolving viscoelastic material with recovery kinetics—rather than as a time-invariant continuum—are more likely to transfer across formulations and sites. Similarly, models that incorporate diffusion-limited curing or dilution penetration can explain why printed gels exhibit internal gradients that drive anisotropic failure. The practical goal is not perfect prediction but rather identifying robust regions within the process and formulation space where gel-state variables remain within safe bounds despite realistic disturbances. This approach aligns with the quality-by-design mindset and is especially suitable for translation into clinical care settings.

### 4.5. Clinical Endpoints and Translational Validation: Building Evidence That Is Gel-Specific and Patient-Centered

Ultimately, IDDSI compliance and instrumental metrics serve as proxies. Successful translation into clinical practice depends on outcomes that matter to patients and care systems, including safety, intake, effort, comfort, and nutritional status over time. For gel-based printed foods, a significant challenge is that patient experiences can vary widely even within a single IDDSI category. Factors such as xerostomia, fatigue, sensory changes, and comorbidities can influence how a gel bolus is formed and cleared. A gel that meets mechanical compliance may still be rejected if it is sticky, leaves residue, or lacks sensory appeal. Conversely, a gel that feels pleasant may still be unsafe if it fragments under extension or along printed seams. Therefore, translational validation must link gel design variables and process histories to patient-centered outcomes through intermediate mechanistic measurements, rather than treating clinical response as an isolated outcome that is difficult to interpret.

Personalization is likely to become a central aspect, and gel platforms are well-suited for this purpose, as network properties and lubrication can be continuously adjusted, while printing can tailor geometry, portioning, and spatial gradients. The key limitation is not the ability to print a customized shape but rather the capacity to translate an individual’s functional constraints into gel-state specifications with known safety margins. This requires rapid assessment methods that characterize swallowing capacity in a way that can be mapped to gel mechanics, followed by validated workflows that reliably produce the targeted gel state across sites. Building this bridge is an ecosystem-wide task, involving shared testing methods, reference gel materials, and clinical workflows that treat printed gels as manufactured products with defined critical quality attributes. If this ecosystem matures, gel-based additive manufacturing can transition from novelty to dependable clinical nutrition, where the value proposition extends beyond visual appeal to include measurable reductions in variability, improved intake, and safer, more comfortable swallowing experiences.

## 5. Product Design and Application in Older Adults and Dysphagia: A Gel-Centered View

### 5.1. Recognizability as a Gel–Geometry Co-Design Problem

Reshaping texture-modified foods into visually recognizable forms is often seen as one of the most immediate clinical benefits of food printing, with 3D printing being highlighted as a strategy to enhance the attractiveness and realistic appearance of dysphagia-oriented foods [114]. For older adults, recognizability can reduce stigma, support appetite through familiar cues, and restore a sense of autonomy by making meals appear less medicalized. Researchers have noted that more appealing forms can improve both the appeal and potential intake of such foods [115]. However, in gel-based dysphagia products, appearance should not be treated as an endpoint pursued independently of the mechanical properties. Recognizability is a design variable that directly links gel network integrity, water management, and failure modes because formulation and rheology strongly influence the quality of the printed structure and its mechanical behavior. Features that signal identity, such as sharp edges, thin protrusions, embossed textures, or layered color patterns, often create localized stress concentrations under handling and oral deformation. These features can also accelerate moisture loss during warm holding, intensify thermal gradients during reheating, and amplify curing gradients in triggered gels, collectively increasing the likelihood of brittle shells, hard zones, or interlayer seam failures that are not immediately visible. These challenges have been highlighted in broader studies of dysphagia food texture, water management, and printing quality issues [116].

A gel-focused product strategy, therefore, reframes “recognizable shape” as a constrained optimization problem. Geometry should be selected to achieve recognition with the fewest necessary high-curvature features, while gel formulation and setting methods should be chosen to maintain a ductile, cohesive failure mode after realistic service conditions. This is because the mechanical properties of printed foods depend on both formulation and process parameters [117]. This reframing changes how product success should be evaluated. Instead of only reporting preference scores, research should quantify the minimum set of visual cues required for recognition, then translate those cues into mechanically safe geometries that ensure bounded local strain under expected mouth and hand interactions, thus acknowledging the known gap between visual appeal and functional swallowability in dysphagia food research. In practice, this approach implies designing shapes with rounded transitions, avoiding thin unsupported ribs, and favoring macro-scale contours over micro-scale embossing when warm holding or dehydration is likely. It also necessitates selecting gel networks with sufficient fracture energy and recovery kinetics to resist crack initiation at geometric discontinuities, as mechanical and rheological characterization is essential for printed food performance under real conditions [118].

Additive manufacturing offers a unique advantage in this context because it enables the co-programming of the gel microstructure and geometry. For example, if a product requires superficial detail for recognizability, the gel in that region can be designed with a more dissipative network, such as a mixed gel with a broadened relaxation spectrum, while the interior remains softer for improved swallowability. This spatial design is only feasible if it does not introduce interfaces that delaminate or create locally stiff interphases. Consequently, recognizability should be viewed as a comprehensive gel engineering problem for the whole construct, rather than a mere surface styling choice.

**Figure 4:**
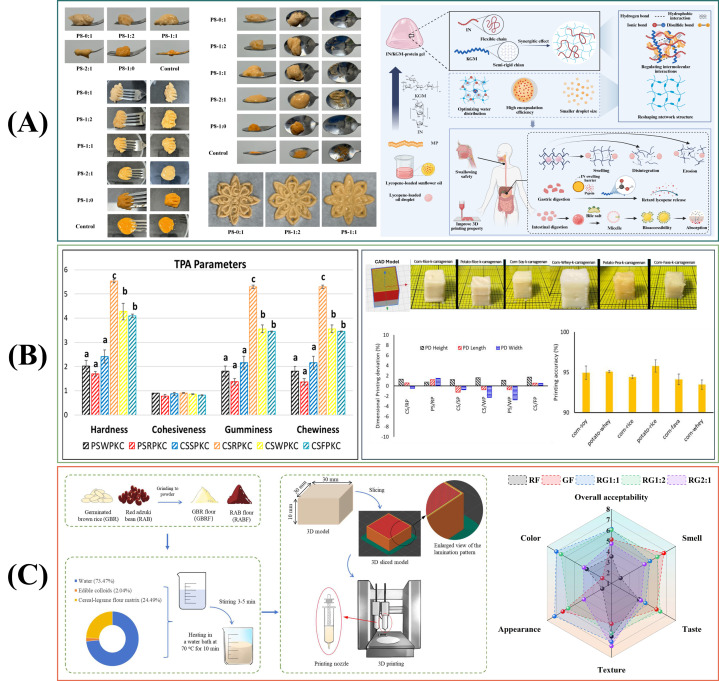
(**A**) IN/KGM formulation that optimizes the gel structure and digestive performance for dysphagia [119]. (**B**) Starch-based gels with hydrocolloids for improved printability and structural fidelity (a–c represent the significance analysis of the data) [120]. (**C**) Cereal-legume starch gels that enhance 3D printability and sensory acceptance [121].

Figure 4 shows three representative studies that explored gel formulation optimization for the 3D printing of dysphagia-friendly foods, emphasizing the integration of texture, printability, and digestive behavior. The first study (Figure 4A) highlights the synergistic effects of inulin and konjac glucomannan (IN/KGM) on the gel network structure and bioavailability, demonstrating how formulations can improve both swallowing safety and print precision. The second study (Figure 4B) focuses on starch-based gels with proteins and hydrocolloids, emphasizing how rheological and mechanical properties affect print fidelity and structural integrity. The third study (Figure 4C) investigates cereal–legume starch blends, revealing the importance of formulation ratios for enhancing printability and sensory acceptance. These works collectively underscore the need for a holistic design approach, balancing recognizability with mechanical integrity and digestive responsiveness, which is crucial for developing personalized, functional dysphagia diets. Further refinement is needed to optimize interface compatibility between gel components and to address challenges in ensuring consistent performance across diverse consumer needs.

### 5.2. Handheld Formats, Autonomy, and the Dual Constraint of Handling Versus Swallowing

Handheld formats, such as finger foods and single-bite portions, address a translational bottleneck often more limiting than texture compliance. Many older adults, particularly those with cognitive impairments or reduced dexterity, benefit from foods that facilitate self-feeding without the need for complex utensils or caregiver assistance. In this context, the potential of gel-based printing extends beyond shape control to the ability to engineer a soft solid that is robust during handling yet deforms safely during oral processing. The technical challenge lies in the fact that handling and swallowing requirements often conflict. A gel that is stiff enough to be grasped and transferred may become too firm or adhesive at the point of swallowing, especially after surface dehydration during holding. Conversely, a gel designed for easy oral breakdown may smear on fingers, stick to packaging, or fracture during transfer, undermining autonomy and creating additional mess.

A gel-centered solution is to treat handheld dysphagia foods as multi-timescale materials. At short timescales and low strains during grasping, the product should behave as an elastic-dominant gel with sufficient yield to resist collapse. At longer timescales and under saliva dilution, it should transition into a lubricious, cohesive bolus that clears with minimal residue. This approach suggests prioritizing gels with strong thixotropic recovery and controllable yielding properties, rather than solely targeting low hardness. Granular microgel systems, emulsion gels, and mixed networks are particularly attractive, as they can provide high yield stress for handling while maintaining a creamy, low-stick perception when microstructural elements are soft and lubricating. However, their benefits are contingent upon stability under repeated shear and thermal cycling, conditions commonly encountered in institutional workflows.

From a translational perspective, handheld food design also necessitates a better definition of target users and safety margins. Products intended for frailty prevention and those for clinically diagnosed dysphagia do not share identical constraints, and conflating these groups can lead to ambiguous performance claims. For dysphagia, the relevant objective is controlled deformation without fragmentation under combined shear-compression and dilution, rather than simply ensuring “softness.” Therefore, evaluation should incorporate handling mechanics—such as grasp stability and transfer integrity alongside oral processing analog tests that include dilution kinetics. If a product passes IDDSI checks but crumbles when picked up, it fails in practice. If it handles well but becomes sticky after warming, it also fails. A rigorous product development pathway must integrate handling performance, serving-time texture after realistic holding, and swallow-relevant failure modes into a single acceptance envelope. In a gel-focused journal framing, the core contribution is to define how gel network design can reproducibly deliver this acceptance envelope across different facilities.

### 5.3. Multicomponent Meals and Personalization as Gel-State Specification Management

Multi-material printing is often promoted as the pathway to personalized nutrition for older adults, and this promise is credible in principle. Printing allows for precise control over portion size, nutrient distribution, and sensory cues, and it can integrate components that would be difficult to assemble consistently by hand. However, multicomponent meals introduce a deeper, often overlooked, complexity. Whole-product compliance is not guaranteed by the compliance of individual components. Each component may meet an IDDSI target independently, but the assembled construct can fail due to interfacial delamination, water migration, differential setting kinetics, and spatial gradients created by triggered curing or thermal setting. These challenges are particularly pronounced for gels, as gel networks are sensitive to water activity, ionic strength, and thermal history. Furthermore, interfaces between dissimilar gel phases often act as preferential crack paths under gentle oral deformation.

A more realistic view of personalization is that it extends beyond nutrient dosing; it involves managing gel-state specifications across the entire preparation chain. A personalized meal must remain within an acceptable mechanical and tribological envelope at the time of service, after storage, and reheating while accounting for expected variability during preparation. This requires embedding personalization within a quality framework that not only defines geometry but also specifies process parameters and post-processing instructions tied to gel-state variables. In practical terms, a “digital recipe” should include deposition parameters that control the shear history, thermal setpoints or trajectories that regulate setting kinetics, as well as holding and reheating protocols that minimize moisture loss and network aging. Without such file-to-food traceability, personalization remains more of a demonstration than a deployable practice.

A gel-centered research agenda for multicomponent products should prioritize engineered interphases. Instead of assuming that two gels will bond adequately when co-printed, interfaces should be designed as controlled transition zones with matched setting kinetics, compatible water activity, and tuned adhesion that resists delamination without creating locally stiff regions. This may involve using bridging polymers, microgel-mediated interfaces, or graded compositions that vary network density smoothly. The scientific objective is to prevent sharp mechanical discontinuities, which often initiate fractures, while maintaining overall softness. Importantly, evaluation should be conducted at the whole-construct level under service-relevant conditions, since interfacial weakening and water migration typically develop during storage and reheating, rather than immediately after printing. Establishing such whole-construct protocols would represent a significant translational contribution and aligns with the expectation of *Gels* for mechanistic clarity in network behavior.

### 5.4. Protein-Forward and Plant-Based Gel Products: Decoupling Nutrition from Brittleness and Residue

Protein enrichment is essential for many older adults due to risks associated with sarcopenia, frailty, and inadequate intake. However, high-protein formulations often destabilize printability, increase firmness, and introduce brittle or crumbly failure modes, especially in plant-based systems where proteins can aggregate heterogeneously and form particulate networks. From a gel perspective, the key constraint is not the nominal protein content but the swallow-relevant fracture and residue behavior after dilution. A protein-rich gel that fractures into granules or forms chalky residues can reduce intake and increase risk, even if it appears compliant in basic tests. Crosslinking strategies can enhance shape retention and reduce slump, but they also tend to narrow the ductile regime, potentially increasing brittle fracture and mucosal coating. This results in products that succeed visually but fail during oral processing.

A forward-thinking gel strategy aims to decouple nutrition delivery from bulk network stiffness. Instead of forcing a uniformly stiff continuous protein gel, protein can be embedded in microstructured formats that maintain a soft macroscopic response. Emulsion gels can distribute protein at interfaces or as structured droplets, enabling high energy density and lubrication while avoiding excessive bulk stiffness. Protein microgels or particulate gels can be engineered as soft, deformable building blocks that jam into a yield stress material with a creamy mouthfeel, provided that particle size, surface hydration, and interparticle adhesion are controlled to avoid graininess and residue. These approaches align naturally with gel science because they treat protein not just as a network former but as a modular structural element whose role can be fine-tuned.

Plant-based dysphagia products also face a common misalignment of goals. Many development efforts implicitly aim to replicate “meat-like” mechanics. However, for older adults with dysphagia, mechanical mimicry is not the primary objective. The goal is predictable, cohesive deformation with low residue under dilution and gentle loads while maintaining enough sensory cues to ensure acceptance. A gel-centered success metric therefore prioritizes the failure mode and lubrication over high firmness or a fibrous bite. This perspective also opens the door for sensory engineering, where aroma retention, flavor release, and visual cues can be optimized alongside gel mechanics. Sensory dissatisfaction, rather than formal texture noncompliance, is often the cause of refusal and low adherence.

### 5.5. Functional Gel Products and Integration into Institutional Service Pathways

Functional dysphagia foods, such as those incorporating probiotics, fiber enrichment, and bioactive delivery systems, align with broader geriatric health goals. However, functionalization often conflicts with gel stability and texture control because the addition of ingredients can alter water binding, ionic strength, pH sensitivity, and interfacial properties, thereby affecting setting kinetics and lubrication behavior. In gel systems, even small compositional changes can influence junction zone density, relaxation times, and syneresis propensity. Probiotic viability may be compromised by thermal exposure, oxygen, and shifts in water activity, while matrices engineered for mechanical stability could inadvertently restrict ingredient release and alter bioavailability. Consequently, the successful incorporation of an ingredient should not automatically be equated with meaningful functional delivery. For gel-based products, function must be evaluated alongside serving-time gel-state constraints, as a functional claim is irrelevant if the texture deviates from safe or acceptable limits in real-world workflows.

Additionally, the greatest barrier to adoption is often the workflow, rather than the formulation. Institutional foodservice operations require sanitation-compatible production, batch consistency, labeling, cold-chain management, distribution, reheating, and point-of-service verification. Gel-based printed foods introduce additional sensitivities, including moisture migration during holding, network aging during storage, and potential heterogeneity from triggered curing. A translationally defensible pathway must, therefore, treat the service chain as an integral part of the product design space. Packaging and reheating protocols should be co-designed with the gel network to control water activity and thermal gradients, thus reducing the likelihood of brittle shells, stiff zones, or surface tack. In practical terms, centralized production using standardized digital files and validated recipes may be the most realistic near-term approach, provided that post-processing instructions are incorporated and that quality checks confirm the intended gel-state at serving conditions.

For research into deployable practice, application studies should not only demonstrate that a product can be printed but also show that it can be reliably manufactured and served within defined control limits. This requires identifying critical control points, such as the maximum warm-hold time before surface dehydration induces tack, reheating profiles that avoid rapid skin formation, and storage durations beyond which gel aging alters failure modes. It also entails reporting acceptable variability ranges for key gel-state variables and linking them to patient-centered outcomes such as intake, satisfaction, and safety proxies. Systems-level validation of this nature is still rare, but it is precisely where gel science can contribute. By treating gel networks as engineered materials with defined critical quality attributes and controlled evolution, over time, the field can move beyond artisanal outputs to create dependable clinical nutrition products that improve both dignity and measurable health outcomes.

## 6. Moving into Real Clinical Use

### 6.1. Keeping High-Moisture Printed Gels Safe

Clinical deployment shifts the focus from whether a gel can be printed to whether printed gels can be produced, held, and served with controllable risk. High-moisture gels, which are often near neutral pH and nutrient-rich, are inherently permissive for microbial growth. Printing can amplify exposure by increasing the surface area, extending the handling time, and introducing additional contamination pathways through cartridges, tubing, and nozzles. Safety cannot, therefore, be an afterthought added after rheology and texture optimization; it must be embedded into the gel platform design and operating window.

A gel-centered perspective clarifies why generic food safety assumptions do not apply to printed dysphagia foods. Different gel types foul and retain residues in distinct ways. Polysaccharide gels can leave hydrated films that persist in crevices. Protein and mixed gels can adsorb and denature on warm surfaces. Multiphase gels may create hydrophobic deposits that resist water-based rinsing. These residues increase the likelihood of biofilm formation if cleaning is incomplete. Additionally, warm extrusion and staged gelation strategies may extend the time gels remain in temperature ranges conducive to microbial growth unless cartridge residence time and pause handling are tightly controlled. For translation, hazards must be mapped specifically to each gel type and workflow. This mapping should drive printer and recipe-specific Hazard Analysis and Critical Control Point (HACCP)-style controls, including defined maximum hold times in delivery systems, validated cleaning and sanitation protocols for small channels and joints, and time–temperature controls that align with the intended service pathway.

Printed gels also introduce geometry-driven risks that traditional cook–serve workflows rarely face. Layer seams, internal voids, and porous architectures can trap moisture and nutrients, creating microenvironments that promote microbial growth and complicate heat penetration during reheating. Triggered gels add another layer of complexity. Diffusion-limited curing can create stiff skins and softer cores, which affect mechanical safety and create cleaning challenges when residues harden in place. For vulnerable and immuno-compromised populations, it is prudent to treat pathogen persistence as a structured risk assessment problem across the entire product chain, including printing, packaging, cold storage, distribution, reheating, and point-of-service verification. Safety evidence collected only at time zero systematically underestimates the risk in real institutional settings.

### 6.2. Ensuring Texture Stays Right Until Serving Time

For dysphagia foods, the relevant quality endpoint is not print success at time zero, but rather a safe and consistent texture at the point of consumption after storage, transport, and reheating. Gel-based products can fail in ways that are not visually apparent. Starch-containing gels can become firm through retrogradation and water migration. Protein gels can synerese or aggregate further, shifting toward brittleness. Emulsion gels can coalesce or undergo phase migration, altering lubrication and residue behavior. Biphasic gel architectures can shift because different phases age at different rates. These changes can push products across clinically meaningful texture boundaries even if their shape appears unchanged. Stability testing should therefore be treated as product qualification, with tests that replicate realistic thermal cycling, warm holding, and reheating profiles used in the target care setting.

Quality management for printed clinical foods differs from conventional operations because the recipe is partially digital [122,123]. Geometry files, toolpaths, curing schedules, and thermal setpoints contribute as much to the structure as the ingredients themselves. Version control and re-validation are thus central concerns. A small change to a toolpath can alter the shear history and layer time, which in turn can affect recovery, interlayer fusion, and anisotropy. A minor modification in curing exposure can shift gradients and fracture pathways. Therefore, clinical translation requires a quality-by-design mindset with defined critical quality attributes and linked critical process parameters. These attributes should be interpretable at serving conditions, such as IDDSI compliance, bounded adhesiveness and residue propensity, and nutrient delivery within tolerance. Critical process parameters should include residence time in the delivery system, the thermal trajectory, curing exposure, and post-processing instructions that staff can reliably follow.

Allergen management and dietary restrictions further tighten the design space and directly influence gel selection. Reliance on dairy, egg, or soy proteins can conflict with clinical constraints, while polysaccharide-heavy gels may compromise protein density unless fortification is engineered in a structurally neutral manner. Modularity is a practical strategy for translation. A robust gel scaffold can maintain consistent mechanical behavior while nutrition is tuned through controlled phases, such as stabilized emulsions or soft protein microstructures that minimize disruption to gel mechanics. Regulatory considerations, including labeling accuracy, shelf-life determination, and compliance expectations for foods intended for vulnerable populations, will ultimately determine what can be deployed, even when technical performance is strong.

Several disturbance factors may shift a printed gel outside its intended swallowing safety window. The most influential are temperature fluctuations and reheating history, as they can alter gel stiffness, phase stability, and lubrication behavior; storage-related viscosity drift, including retrogradation, syneresis, and water migration; saliva dilution and oral residence time, which modify bolus cohesion and interfacial lubrication; and printing-induced heterogeneity, such as interlayer seams or curing gradients. Among these variables, thermal history and saliva dilution are likely first-order factors because they can rapidly change both bulk rheology and bolus integrity at the point of service. Identifying and controlling these disturbances is therefore essential for maintaining swallowing safety in real clinical workflows.

### 6.3. What the Field Should Focus on Next

The most persistent technical gap is the under-specification of printability. Many studies demonstrate that an object was printed, but they do not define the acceptance criteria, quantify variability, or specify which failures are clinically unacceptable. Without shared metrics for filament stability, interlayer adhesion, defect tolerance, and serving-time texture drift, the results remain confined to laboratory settings. For gel platforms, the key shift is to define printability as a reproducible gel-state outcome across the entire service chain, rather than as a one-time geometric success.

The next phase of research should strengthen the mechanistic link between categorical compliance and real oral processing. While the IDDSI is essential, it does not uniquely determine residue or failure mode under combined shear, compression, extension, and dilution. Gel research can provide this bridge by integrating time-dependent rheology, fracture behavior, and tribology under saliva-like dilution and realistic post-processing conditions. Scale-up and cost will also play a significant role in adoption. The most impactful advances may come from system integration: tolerant gel formulations, washdown-ready printers with metered deposition, and embedded quality checks that confirm the gel state at serving conditions rather than assuming it. If the field moves in this direction, gel-based printed foods can transition from impressive demonstrations to dependable clinical products that improve safety, intake, and everyday care workflows.

## 7. Conclusions

This review underscores the transformative potential of gel-based 3D food printing for dysphagia management, emphasizing the integration of personalized nutrition with safe, swallowable textures. By leveraging additive manufacturing, we can address the limitations of traditional texture-modified diets, such as inconsistent textures and diminished appeal. Gel-based inks, including polysaccharides, proteins, and multiphase systems, offer precise control over mechanical properties and nutrition, enabling tailored solutions that can be optimized for both printability and swallowing safety. Advances in thermal management, interlayer bonding, and post-print curing processes facilitate the development of gels that maintain structural integrity during printing while providing safe, easy-to-swallow products for patients with dysphagia. Tools like the IDDSI for texture classification serve as a critical bridge between food engineering and clinical requirements, thus ensuring that printed foods are both functional and safe. However, challenges persist in ensuring reproducibility, long-term storage stability, and consistent performance under varying conditions. Future research should focus on developing standardized testing protocols, enhancing multi-material interface bonding, and incorporating real-time monitoring systems to optimize print quality and texture evolution. These advancements will help translate innovations into clinical practice, ultimately improving patient outcomes.

## Figures and Tables

**Table 1:** Emulsion-based and particle-stabilized inks for extrusion 3D printing of dysphagia-oriented foods.

Ink and Interface Strategy	Key Measurements Reported	Swallow Relevant Outcome	Limitation	References
Protein emulsion gel; interfacial network tuned by the SPI:EWP ratio	Bulk rheology and texture, print performance, and microstructure	IDDSI Level 5 achieved at an optimized SPI:EWP ratio	Limited saliva dilution and tribology; oil release under oral shear not deeply tested	[54]
HIPE gel inks; PPI–inulin complexes build dense interfacial barrier and droplet packing	Thixotropic recovery, cryo-SEM, and thermal stability	IDDSI evaluation in an elderly cohort and classified as transitional foods (Class IV and V)	Interfacial rheology rarely measured directly and limited mechanistic mapping to bolus cohesion and residue	[49]
Emulsion filled gels vs. HIPEs; structure built by reversible H-bonding vs. droplet jamming	Thermoreversibility, shear thinning and recovery, printability index, and height maintenance	Establishes a low-fat route to high-fidelity printing via thermoreversible emulsion gels	Swallow metrics (IDDSI, lubrication, and residue); oral stability under dilution not standardized	[55]
Emulsion as a texture modifier and lubricant inside a protein gel matrix	Rheology, texture, friction force, and IDDSI tests	Reduced friction force and chewiness; IDDSI Level 5 (minced and moist)	Needs a clearer link between droplet size and lubrication vs. residue risk; storage and reheating effects under-explored	[53]
Starch-based emulsion gel; glycerides modulate network and structural precision	Print precision metrics and texture control	Designed explicitly for texture-controlled dysphagia printing	Often missing tribology and saliva dilution protocols needed for predicting residue and bolus breakup	[56]
Pickering emulsion gels stabilized solely by deformable SPI microgel particles	Droplet packing and gel strength, viscosity and G′, print accuracy, and stability	Proposed as a platform for the elderly and those with swallowing disorders, with high shape accuracy	Mouthfeel and graininess risk not systematically assessed; interfacial viscoelasticity not directly quantified	[57]
Pickering emulsion gel stabilized by protein–polysaccharide hybrid particles	Particle size and zeta potential, gel strength and viscosity, and print self-support	Demonstrates stable extrusion and self-support in a high-oil-content Pickering gel	Dysphagia outcomes not targeted; lubrication vs. residue and oil exudation under oral shear not tested	[58]
O/W Pickering emulsion gels; noncovalent interactions tuned by polysaccharide charge and pH	Recovery rate and dimensional deviation	Identifies formulations with low deformation and high recovery for printing	Lacks swallow-centered validation (IDDSI, saliva dilution, and tribology); thermal cycling stability not emphasized	[59]
Pickering emulsion gels improved by regulating protein–polysaccharide electrostatics	Print performance and rheology	Positions Pickering gels as tunable inks for structured foods	Limited tribology and saliva dilution. The predictive link from an interfacial state to filament fusion and bolus cohesion remains weak	[60]

**Table 2:** Thermal history and setting kinetics in gel-based 3D food printing.

System/Focus	Thermal/Setting Variable	Key Findings	References
Gelatin/gelatin-pectin/gum mixtures	Cooling-induced gelation and rheology	Quantified G′, yield stress and phase angle effects on shape retention and fidelity; implicates thermal history in post-extrusion stability.	[75]
κ-Carrageenan and agar gels	Thermal transitions	Thermal transitions affect network formation and printability; printed gels show layering and delamination indicative of poor interlayer bonding.	[76]
Inulin-based emulsion gels	Temp scanning and freeze–thaw cycling	Elasticity and print integrity vary with thermal exposure; peak moduli at low T show that thermal history alters network rigidity.	[77]
Corn starch–salmon protein gels	Post-processing thermal treatments	Freeze drying vs. oven drying affect dimensional stability and texture, tying thermal history to final gel fidelity.	[78]

**Table 3:** IDDSI plus mechanistic evidence used to qualify 3D-printed gel-like dysphagia foods.

System or Target Food	IDDSI Use (Level, Test)	Mechanistic Add-Ons Beyond IDDSI	Key Gap or Limitation	References
3D-printed pork with hydrocolloids	IDDSI alignment reported transitional texture targets.	Texture profile, rheology, and microstructure links to gum blends.	Limited capture of printing-induced anisotropy and interlayer defects.	[97]
Fresh vegetable-based inks	IDDSI tests used to judge dysphagia suitability.	Rheology, texture, microstructure, and printability mapping.	Passing the IDDSI does not guarantee stable oral lubrication after saliva dilution.	[98]
Multi-ingredient nutritious meals	Dysphagia suitability assessed with the IDDSI.	Hydrocolloid optimization. Print fidelity vs. structure.	Limited clinical realism. Heating, holding, and service chain variability under-tested.	[6]
Emulsion gel-based printable inks	Dysphagia IDDSI assessment reported.	Emulsion gel rheology engineering for extrusion stability.	The IDDSI alone weakly discriminates between gels with different yields and recovery kinetics.	[49]
HIPE gel prints for dysphagia	IDDSI plus sensory chewing and swallowing tests.	Rheology. Microstructure, oral suitability and digestive fate.	Sensory protocols vary. Hard to generalize across care settings without standard histories.	[99]

## Data Availability

Not applicable.

## Abbreviations

IDDSI	International Dysphagia Diet Standardization Initiative
SPI	Soy protein isolate
DSC	Differential scanning calorimetry
HIPE gels	High-internal-type emulsion gels
EWP	egg white protein
PPI	Pea protein isolate
HACCP	Hazard analysis and critical control point
